# Objective structured clinical examination: Challenges and opportunities from students’ perspective

**DOI:** 10.1371/journal.pone.0274055

**Published:** 2022-09-02

**Authors:** Nazdar Alkhateeb, Abubakir Majeed Salih, Nazar Shabila, Ali Al-Dabbagh

**Affiliations:** 1 Department of Medical Education, College of Medicine, Hawler Medical University, Erbil, Iraq; 2 Department of Community Medicine, College of Medicine, Hawler Medical University, Erbil, Iraq; 3 Faculty of Nursing, Tishk International University, Erbil, Iraq; Ege University Faculty of Medicine, TURKEY

## Abstract

**Background:**

Objective structured clinical examination (OSCE) has been used in evaluating clinical competence in health professions education around the world. Despite its implementation in Iraq for around a decade, limited studies investigated the challenges and opportunities to improve the standard and quality of this examination from student’s perspective.

**Methods:**

This qualitative study was based on an online open-ended questionnaire survey that was carried out in the College of Medicine, Hawler Medical University, Iraq at the beginning of the 2018–2019 academic year. A convenience sample of 180 students in the clinical phase (4^th^, 5th, and 6^th^) year of study were invited to participate.

**Results:**

A total of 141 students responded to the online questionnaire. The participants were generally happy with the OSCE, and they recognized many positive aspects, including the role of the OSCE in increasing confidence, engagement and motivating learning, the role of the OSCE in achieving a higher level of learning, the content validity of the OSCE, and the quality of the OSCE. The main weak points of the OSCE identified by the students included unfairness, gender discrimination, duration of the OSCE, and the behavior of the examiners. Suggestions to improve the OSCE examination included improving the examiners’ behavior, with the focus on the training of the examiners, and avoiding discrimination among students.

**Conclusions:**

Most of the students were generally satisfied with the current OSCE examination. The main concern of the students was related to the organization of the OSCE. Valuable suggestions were raised to improve the OSCE quality including examiners’ and simulated patients’ training.

## Introduction

Objective structured clinical examination (OSCE) is an assessment method developed by Harden and his colleagues to evaluate the candidates’ clinical performance accurately. It aims to assess competence at the performance level of “show how” based on Miller’s competency pyramid [1, 2].

The OSCE has been used in assessing clinical competence in health professions education globally. The experiential aspect of training necessitates placing more emphasis on effective evaluation of students’ performance in practice settings bearing in mind the complexities of competencies tested at various OSCE stations may differ significantly [3].

Measuring an attitude is a hard task, as it has to be measured indirectly. One cannot see the attitude directly; it must be measured from what the individual says or does. There are different methods of measuring attitudes. A number of those are very simple, while others are complicated. Broadly, attitudes can be measured by self-report methods, attitude scales, and involuntary behavior methods [4].

Acceptability is an important component of an assessment involving several aspects; cost-effectiveness, cultural acceptability, time constraints, and feasibility. Acceptability involves several stakeholders; teachers, administrators, patients, the public, and, most importantly, students [5, 6].

In Iraq, the final year medical students’ assessment was composed of a two-part assessment; advanced clinical competence that assesses the skills and a written component. Like many developing countries, Objective Structured Clinical Examinations (OSCE)were only introduced to most medical schools in the last decade. At its beginning, it was combined with other forms of clinical assessments [7]. It was conducted at consecutive days, with different specialties (i.e. OSCE in medicine, surgery, pediatric, obstetrics and gynecology separately). Different formats of the OSCE have been tried since its first introduction at Hawler Medical University in 2007 including addition of global rating to the checklist, using blueprint for ensuring the content validity and make it a comprehensive clinical exam that assess the clinical competence in all specialties in one day [8]. Many studies evaluated OSCE from both student and examiners perception [7, 9]. However, most of them based on quantitative methods. This study aimed to assess the students’ perspectives about the challenges face OSCE, and how to improve its quality using qualitative method.

## Methods

### Study design

This qualitative study was based on an online open-ended questionnaire survey.

#### Setting and time

This study was carried out in the College of Medicine, Hawler Medical University, Erbil city, Kurdistan Region of Iraq at the beginning of the 2018–2019 academic year.

#### Participants

The population included students in the clinical phase(4^th^, 5th, and 6^th^ year of study).

#### Sampling method

Convenience sampling was used to select 60 students from each of the 4th, 5^th^, and 6^th,^year. The participants were invited electronically to fill a google form about their experiences and perceptions of the OSCE in the College of Medicine.

A revised form of the questionnaire was prepared using the google form and was sent by email to 180 participants, 60 students from each of the 4^th^, 5^th^, and 6^th^ years.

#### Data collection

The questionnaire was designed by authors after extensive literature review [3, 10] and comprised of items to gather demographic data from the respondents regarding their gender and year of the study, the second part of the questionnaire addressed the students’ view on the OSCE by answering three open-ended questions evaluating the OSCE stations in terms of strength, weakness, and recommendations for improvement.

Google form had been used to overcome the problem of incomplete response to questionnaire benefiting from required feature of questions in the google forms.

#### Data analysis

Descriptive statistic was used to analyze the demographic characteristics of the participants. The qualitative data that was generated from the open-ended questions were analyzed using thematic content analysis. Interpretable responses were summarized and categorized into themes.

The qualitative data analysis comprised of a thematic analysis of the answers using common coding techniques through reading the comments and identifying the main themes within these comments which were done by the first two authors. Using these identified themes, a structured classification of codes was generated. The data were coded manually in a series of repetitive steps and the code structure was reviewed and refined multiple times as new insights were developed, and new relationships between the themes present in the comments were extracted. Each open item was analyzed and reported independently. More than one response was coded for each subject when necessary. Duplicate answers were only coded once. Illegible, blank, and off subject answers were coded as missing data. The number of responses for each theme were calculated and presented for each question in the result.

#### Trustworthiness

Trustworthiness is the level of adequacy or soundness in qualitative studies [11]. Ensuring a qualitative study’s trustworthiness involves important steps such as describing data analysis and justifying the reliability of the gathered data [12]. Ensuring the trustworthiness in this study involved considering the field experts’ comments, creating a good relationship and obtaining the student’s trust, using suitable time and place for the data collection, and reading the transcripts several times. Moreover, the researchers’ long-term teaching experience helped ensure their reliability.

### Ethical consideration

This research was approved by the Ethics Committee of College of Medicine, Hawler Medical University (Reference code 6/3 on 23/6/2020). The participation was voluntary and anonymous. Informed written consent was obtained electronically from all the participants with detailed information on the study.

## Results

From a total of 180participants chosen, 141 responses were received. The response rate was78.3%, forty-nine students were male 49 (34.7%) while 92(65.7%) were female. The received responses were48, 42, and51from 4th, 5^th^ and 6th year students respectively, as shown in Table 1.

**Table 1:** Demographic distribution of the sample.

Variable	No.	Percentage
Gender		
Male	49	34.75
Female	92	65.7
Year of the study		
4^th^ year	48	34
5^th^ year	42	29.78
6^th^ year	51	36.17
Total	141	100

### The positive aspects of the OSCE

The participants were generally happy with the OSCE, and they recognized many positive aspects related to this experience. They emphasized the role of the OSCE in increasing confidence, engagement, and motivating learning and, performed quickly. The study participants identified a number of positive aspects of the OSCE like: making them study practical sessions better and feeling more responsible about their learning, facing real examples of their future life and working under pressure which will help them to overcome their future’s challenges.

Examples of the relevant quotations included:

Student #38 ’*Makes you study the practical sessions better*, *you feel more responsible and feel like a doctor*.’

Student #64 ’*We face what is important and necessary for us to know in the future in real life and get evaluated on it*.’

The participants emphasized the role of the OSCE in the higher level of learning.

Examples of the relevant quotations included;

Student #95 ’*What I like most about it is that it is testing how well the student can function under pressure*, *and you cannot perform well by memorization*, *you must have understood the knowledge*. *It also prepares us for any international exams we might face in the future*.’

The majority of students commented on the content validity of the OSCE positively. Examples:

Student #97 ’*Informative and covers the topics*.’

In general, the respondents were satisfied with the quality of the OSCE

Student #78’*It is a new way of examination and prepare us for being up to date*, *and sometimes reflect real scenarios*’

### The weakness of the OSCE

The participants identified a number of negative aspects of this experience. Their main concern was related to the unfairness of their evaluation by their teachers and the teacher’s little experience in this field. Other concerns included time, duration, and organization of the OSCE which are very important elements for the students to pass their assessment successfully.

Examples of the relevant quotations included:

Student#38 ’*Each student is being evaluated by a different examiner; the score is not fair*, *because some examiners are tough and ask more difficult stuff than other lecturers*.’

Student #89’*For the second group of students*, *doctors usually get tired and ignore them*.’

Gender discrimination was another problem raised by some students.

Exampleof quotations included:

Student #68 ’*Gender discrimination is obvious*. *Females are always getting better marks even though the majority of them are less skilled than males*, *double check the marks*!’

Some of the participants complain about the untrained examiners, which affect their performance during the OSCE.

Examples of quotations included:

Student #66 ’OSCE sometimes is highly dependent on the examiner. some of them are mask faced and they want to make OSCE even harder and more stressful. while other groups of students examined by more grateful and friendly doctors’

Another weakness that was also highlighted was the organization of the OSCE. Examples of quotations:

Student #71 ’the delay happens at the time of OSCE due to some shortage of (patient,doctors.etc), it’s nerve breaking’

Student #93’*Students should not hold too long in the waiting room since this long period of waiting causes exhaustion and stress in students*.’

Student #95’*The temperature of the hall we stayed in till our names were called was extremely hot*! *The examination area was ok*, *but the waiting area was too hot to bear*.’

Time was not adequate or short according to station tasks or due to delays in the start of the OSCE, as evidenced by

Student #112 ’*Time is not adequate since we lose some time while changing between stations*.’

Another weakness was cheating; although it was mentioned only in 3 comments, it requires to further focus on it to come up with feasible solutions.

Student #95 ’*Something I would like the authorities to know about is that most of the time*, *the last group of students receive information about the stations by text messages from those who have finished their exams*. *It is very unfair*. *This problem needs to be taken care of*. *I suggest putting someone to watch over the students the entire time*.’

### Suggestions for improving the OSCE

The participants made many suggestions to improve the OSCE. The main emphasis was on improving the behavior of the examiners during the examination, with the focus mainly on the training of the examiners and avoiding discrimination which will help students’ to feel more comfortable during the examination. The other suggestions were related to the time and organization of the OSCE in a way that help students to feel more relaxed and under less pressure.

Examples of the relevant quotations included:

Student#50 ’*Training the examiners so they can assess students objectively*.’

The participants also indicated that the content and organization of the OSCE stations should be revised and that the stations should contain more common and practical skills, and not all examinations should be done in one day.

Examples of the relevant quotations included:

Student #67’*Before putting any station think about the time*, *in most OSCE Examination first group have more time than other groups who do exam after them*, *they decrease the time*.’

To solve the problem of uncooperative patients, some students suggest the type of consent from patients.

Student #38 ’*I strongly recommend patients in our governmental hospitals to fill out a form on admission that allows students to take a history from them (just like a consent form)*. *Then*, *we*, *as medical students*, *will be more comfortable around them*. *This will encourage us to take a history from the patients*. *Now I am annoyed when I am being asked to take a history"* others in favor of having more real patients *"bring up real patients*.’

## Discussion

The OSCE is a standardized format of the examination. The feedback provided by the students is highly valued and helps in the advancement and improvement of the testing process [13].

A strong relationship between assessment and learning has been thoroughly stated in the literature. The OSCE has proved to stimulate and enhance students’ learning in realistic self-assessment [14]. Therefore, it is suggested that the OSCE could be adapted and used as a diagnostic tool to guide student learning [3].

The participants recognized many positive aspects that were related to this experience. They highlighted the role of the OSCE in the higher level of learning. This indicates that the OSCE can serve as a tool to improve students’ learning. This finding was also reported by Alaidarous (2016) although, in their case, the degree of satisfaction on the educational impact of the OSCE was higher. This might be due to the difference in the sample as they studied residents’ perception [15].

A practicing physician should be competent in domains like clinical skills (e.g., history taking or performing a physical examination), practical procedures, patient management, and communication. Helping students in developing the above competencies is a major objective of medical education [16]. The students perceive the OSCE as a reflection of their performance quality. This conclusion can be drawn from students’ positive opinion on the OSCE as a true measure of clinical skills and simulating real-life experience. This result was in accordance with the previous studies [14, 16].

The majority of students commented on the content validity of the OSCE positively. The OSCE has proven to be both a reliable and valid mode of evaluation of clinical skills. It is also generally well accepted by both the students and the faculty worldwide [17].

One of the important issues highlighted by the students was about the organization of the OSCE, like many students examining in the same room, long period of waiting before the examination, and uncomfortable environment during the OSCE. The most likely cause of these issues was implementing the final OSCE in a hospital due to insufficient infrastructure at the college campus.

Uncooperative patients were another problem raised by the students as a weakness of the current OSCE examination. A patient who has been frequently evaluated by a large group of students during an OSCE feels uncomfortable. Additionally, it might be challenging to standardize real patients’ clinical characteristics, which can cause variations in OSCE results for students [18]. Therefore, OSCE requires investment in identifying and training simulated and/or standardized patients well in advance of the assessment [19]. This finding has important implications for developing a systematized training for simulated patients to prepare them more adequately.

Notably, a considerable proportion of students felt that personality and gender affected their scores for this examination. This finding was consistent with Al Nazzawi [20]. However, this issue was studied in another study by authors where the results showed that only seven stations out of 23 stations had a significant difference between males and females, in which six stations were in favor of females and one in favor of males [8]. Similar results were obtained by Schleicher et al. (2017), who conducted a study on five medical schools and tried to answer whether the scoring was biased by student’s gender [21].

The difference in marks was likely caused by the increased examiner strictness over time due to the combination of exposure to more successful students and growing experience [22]. Variation by the time of day has been attributed to examiner tiredness as the OSCE continues, this may explain the difference by the time of day [23]. The same viewpoint was raised by our students.

The greatest perceived attitude toward the OSCE was that the OSCE is very stressful. This was the response of the majority of the students and it is aligned with many studies, which documented that the OSCE can be a strong anxiety-producing experience [19, 24].

One of the important suggestions of the students was to train examiners. Training examiners is vital for both novice and experienced examiners and is an important factor to ensure a valid OSCE. Examiners may use themselves as a reference point, leading to stricter student ratings or they may set higher pass threshold in the OSCE due to their confidence of the grading format or understanding of student standards [23, 25].

Students’ overall acceptance of the instrument and participation in the evaluation were encouraging. Feedback from students was useful in improving the process. It is also sending a clear message to students that the achievement of overall competence is crucial to clinical practice. Eventually, these provide the necessary circle to run the continuum of curriculum development.

This study provides valuable students insights on application of OSCE through using open ended questions that allow better understanding the student’s true feelings and attitudes about the OSCE and try to address the gap in literatures which are concentrated more on quantitative results using Likert scales.

### Limitation of the study

The findings of this study have to be seen in light of some limitations. This study is based on one institution, which might limit its generalizability. Many steps were taken to improve reliability, like keeping all the digital data of the study and detailed description of the methodology in a way that it can be replicated in other institutions. Therefore, the findings of this study can be applicable to a large number of countries with the same circumstances regarding resources and infrastructures, especially in developing and/or low resource countries.

Although the qualitative approach to study the research question was one of the strengths of this study, using one tool, the online open-ended questionnaire, and not including other tools like focus groups and interviews was a considerable limitation. Participation of a large number of students with their opinion through the google form, which was used as a tool for the online questionnaire to overcome the problem of missing data, might not go in line with the sampling strategy of qualitative studies. However, this wide participation helped in having a wide range of opinions and viewpoints.

## Conclusions

In summary, this study has provided a comprehensive understanding of the strength and weaknesses of OSCE through student’s perceptions. Most of the students were generally satisfied with the OSCE examination. The main concern of the students was related to the organization of the OSCE regarding long waiting time for the last batch and some examiners’ behavior in the exam. It also uncovered examiner/simulated patients training gaps and raised a valuable suggestion on the continuous involvement of the students in the process of feedback and development of OSCE quality. The finding of this study will provide a guide for policymakers to concentrate more on faculty development in the field of assessment. Further studies using more qualitative tools, like focus groups and interviews, are needed in the future.

## Supporting information

S1 Data (XLSX)

S1 File (PDF)
