# Supplementary material for: Objective structured clinical examination: Challenges and opportunities from students’ perspective
Source: PLoS One. 2022 Sep 2;17(9):e0274055. doi: 10.1371/journal.pone.0274055 (PMC9439190; doi:10.1371/journal.pone.0274055)
Supplement: S1 File — (PDF) [file pone.0274055.s002.pdf]

OSCE student feedback

Your opinion and experience with us are very important because your feedback will improve the quality of OSCE assessment for future years. It will take less than 5 minutes to complete the survey. All information you provide will remain anonymous and only aggregate information will be shared publicly and with higher education officials.

* Required

1. Gender *

   Mark only one oval.

   - ( ) Male
   - ( ) Female

2. Grade *

   Mark only one oval.

   - ( ) Fourth year
   - ( ) Fifth year
   - ( ) Sixth year

3. Strengths of the current OSCE *

   ____

4. Disadvantages of the current OSCE *

   ____

5. Suggestions to improve the current OSCE *

6. The permission for using information *

Mark only one oval.

- ( ) I give my permission for using this information for research purposes
- ( ) I don't give my permission for using this information for research purposes
